# Sensitivity Enhancement of NMR Spectroscopy Receiving Chain Used in Condensed Matter Physics

**DOI:** 10.3390/s19143064

**Published:** 2019-07-11

**Authors:** Petar Kolar, Mihael S. Grbić, Silvio Hrabar

**Affiliations:** 1 Department of Physics, Faculty of Science, University of Zagreb, Bijenička cesta 32, HR-10000 Zagreb, Croatia; 2 Faculty of Electrical Engineering and Computing, University of Zagreb, Unska 3, HR-10000 Zagreb, Croatia

**Keywords:** NMR, noise, noise figure, amplifier, NMR measurement time decrease, sensitivity enhancement

## Abstract

Assurance of high measuring sensitivity is one of the most challenging issues for any nuclear magnetic resonance (NMR) spectroscopy system. To this end, we propose an accurate noise model of the entire probe-to-spectrometer receiving chain for condensed matter physics, based on the concept of noise figure. The model predicts the propagation of both the signal and noise levels in every component of the NMR spectroscopy receiving chain. Furthermore, it enables identification of the "weakest" component and, therefore, the optimization of the whole system. The most important property of the proposed model is the possibility to find system parameters that reduce the measurement time by an a priori calculation, rather than an a posteriori approach. The model was tested experimentally on several different samples. It was found that the measurement time can still be significantly shortened, down to at least one half of the measurement time, starting from optimized conditions with commercially available components. Thus, the proposed model can be used as a tool for both quantitative analysis of the noise properties and a sensitivity prediction of practical NMR systems in physics and material science.

## 1. Introduction

With the technological development of nuclear magnetic resonance (NMR) systems, the associated measurements can be made with higher precision than ever before. This allows for detailed microscopic studies of samples with very weak response signals that would otherwise lead to notably longer measurement times. Further improvements of measurement accuracy and decreases of measurement time are limited, primarily, by the noise properties of the NMR spectroscopy system [1]. Widely used solutions include the averaging of multiple measurements and cryogenic cooling of the NMR probe, coil, or even the pre-amplifier [2]. Although these approaches are effective, most often the improvements are achieved in a purely experimental way (i.e., by repeating experiments for different sets of system parameters and finally choosing the most appropriate one). Quite often, the maximum improvements and limitations of an existing system remain unknown. Due to this, it may occur that a NMR setup operates in an unoptimized state. Therefore, the overall measurement time may be much longer than the shortest possible time that would be achieved in optimized conditions. Sometimes, even investments into expensive upgrades of the system (e.g., a special low-noise pre-amplifier) cannot increase the system performance substantially, due to the limitations of other system components. While it is relatively easy to estimate the amplitude of the response signal at the probe with the measured sample [3], an a priori prediction of the response signal and associated noise at the chain end (the spectrometer screen) needs a more complex approach. Such an approach should combine knowledges of physics, radio frequency (RF) engineering, and signal processing.

Here, we use the concept of noise figure (widely used in RF engineering [4,5]) and derive a closed-form expression that accurately predicts the noise properties of the most sensitive part of the NMR spectroscopy system—its receiving chain—and enables determination of signal-to-noise ratio (SNR), both at the input and the output of the NMR receiving chain.

There have been several calculations in the literature that estimated the voltage SNR at the NMR coil caused by the response signal of the sample under measurement ([3], further developed in [6] for coils of general shape). We will focus, here, on the case of a solenoid coil of volume $V_{c}$, wound around a sample of volume $V_{s}$ (because of this, the filling factor $\eta =V_{s}/V_{c}$ is approximately 1), where a spin will induce the voltage [3]:

(1) $$U_{spin}=\frac{1}{6}N\frac{\gamma^{3}h^{2}B_{0}^{2}\mu_{0}I\left(I+1\right)r_{c}^{2}n_{c}}{k_{B}TV_{s}}.$$

where *N* is the number of NMR nuclei in the sample, γ is the nuclear gyromagnetic constant, $B_{0}$ is the magnetic field intensity, *I* is the nuclear spin size, $r_{c}$ is the coil radius, $n_{c}$ is the number of turns of the coil, *T* is the sample temperature, and $V_{s}$ is the sample volume. Additionally, *h*, $k_{B}$, and $\mu_{0}$ are the Planck constant, Boltzmann constant, and vacuum permeability, respectively. The mean-averaged thermal noise voltage at the coil terminals reads as:

(2) $$U_{noise}=2\sqrt{k_{B}T\Delta fR_{c}},$$

with $R_{c}$ denoting the coil resistance and Δf the frequency bandwidth. From above, it follows that the SNR equals ${SNR}_{in}^{0}={\left(U_{spin}/U_{noise}\right)}^{2}$. However, (1) overestimates the amplitude of the NMR signal, as it does not take into account the loss of intensity due to short spin–spin relaxation time, reduction of the spectral weight in the case of quadrupolar splitting [7], broadening of NMR line-width due to intrinsic and extrinsic inhomogeneity of the samples, and abundance of the measured isotope [8]. Hence, ${SNR}_{in}^{0}$ needs to be taken just as an initial guess of NMR signal level and applied in the analysis, accordingly. We will, however, use it to check the validity of our expression, which will serve as a more complete and pragmatic counterpart of ${SNR}_{in}^{0}$. To the authors’ knowledge, the enhancements of noise properties, as well as the measurement sensitivity, of NMR systems have been difficult to quantitatively track or predict by a typical user, and such an analysis has not been published so far.

## 2. Noise Figure of NMR System Receiving Chain

### 2.1. General Approach

One of the biggest problems for modern NMR measurements is dealing with the very low response signals of specific samples (where the coil signal is lower than the noise level) [9]. Thus, it is necessary to identify the weak elements of the receiving chain of the NMR system, so that users can check the feasibility of further improvements. These improvements should enhance measurement sensitivity and, consequentially, decrease measurement time.

### 2.2. NMR Spectroscopy System

**Figure 1:**
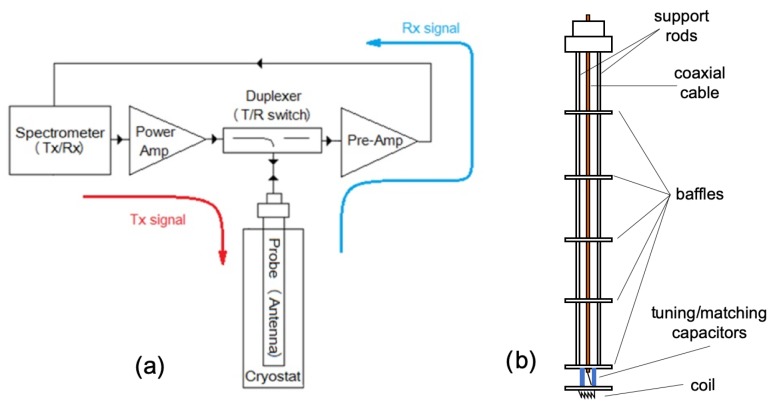
NMR spectroscopy system: (**a**) Basic schematic diagram and (**b**) schematic of the probe used in condensed matter physics (co-axial cable (orange) connected to the coil at the bottom by two variable capacitors (blue)). Thin stainless steel tubes parallel to the cable keep the structure stable, while transverse plates block external radiation from reaching the coil space.

The basic schematic diagram of a NMR spectroscopy system is shown in Figure 1a. This system operates in two modes: Transmitting (Tx) mode and receiving (Rx) mode. In the Tx mode, high-power pulses (up to the order of a kW), used for excitation of the nuclei in the sample, are first generated by a special oscillator. The pulses are amplified by a power amplifier and transmitted, by a duplexer, to the probe coil. These high-power pulses generate a magnetic field within a coil that excites the nuclei of the embedded sample (Figure 1b). The sample response signal has a very low magnitude (on the order of a fW), which is amplified before arriving at the spectrometer’s receiver. Amplification of the signal is achieved by a low-noise pre-amplifier. In the spectrometer, the received signal is down-converted to an intermediate frequency and further amplified by a variable-gain amplifier that allows amplitude optimization before analog-to-digital (A/D) conversion [10]. After detection and A/D conversion, the signal is digitally post-processed using different methods, such as time averaging of multiple measurements and digital filtering, and such a signal is, then, shown on the spectrometer screen [7,11]. In the case of very low levels of the receiving signal (which are close to or even smaller than the background noise level [4]), the sensitivity of the NMR system is often limited. This is why the receiving chain properties need to be carefully analysed and quantitatively defined.

### 2.3. Concept of Noise Figure

To define the noise properties of the NMR spectroscopy receiving chain, we decided to use the concept of a noise figure (*F*) [4,5]:

(3) $$\begin{array}{c} F=\frac{{\mathrm{SNR}}_{in}}{{\mathrm{SNR}}_{out}}{|}_{T=T_{0}}\left[\mathrm{lin}.\right]\Rightarrow 10\cdot \log\left(\frac{{\mathrm{SNR}}_{in}}{{\mathrm{SNR}}_{out}}\right){|}_{T=T_{0}}\left[\mathrm{dB}\right], \end{array}$$

where ${\mathrm{SNR}}_{in}$ and ${\mathrm{SNR}}_{out}$ stand for the ratio of signal power and the root mean square (RMS) value of the noise power at the input and the output of the chain’s element for which we want to define the noise properties, respectively.

**Figure 2:**
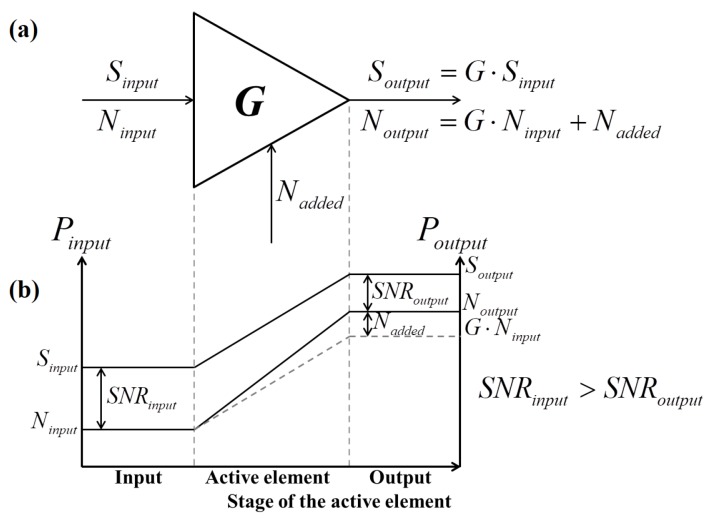
(**a**) RF device schematic diagram and (**b**) signal and noise levels through the device.

The noise figure describes the SNR deterioration of the signal by its transmission through each element of the RF chain. Signal deterioration occurs due to the internal noise generated within all RF elements. Internally generated noise is added to the signal in a given RF system (see Figure 2). When a signal passes through the amplifier, it is amplified by the value of the amplifier’s gain *G*, as is the noise level of the input signal. If the amplifier was ideal, the input noise level would only be amplified by the same value as the input signal and SNR would remain unaltered at the output. However, since the amplifier is not ideal, it adds internal noise, which, together, with the initial noise, causes deterioration of the output SNR.

In addition, (3) is valid only if the noise at the input of the measured element is at the so-called standard temperature $T_{0}=290$ K [4,5]. The value *F* can be defined both for each element of the receiving chain, as well as the entire chain altogether. Generally, when the elements are connected in a cascade, it can be shown that the overall *F* value of a chain can be expressed as [4]:

(4) $$F_{overall}=F_{1}+\frac{F_{2}-1}{G_{1}}+\cdots +\frac{F_{n}-1}{\prod_{i=1}^{n-1}G_{i}}{|}_{\forall Z_{i}=Z_{0}},$$

where $F_{i}$ stands for linear noise figure value of *i*th element after the source (in our case, the NMR coil), while $G_{i}$ stands for the linear value of its power gain. It is important to mention that (4) has been derived under the assumption that the input and output impedances of all elements in the chain are matched to characteristic impedance of the system $Z_{0}=50\;\Omega$ [4]. In other words, (4) presumes that there is no signal reflection between any two neighbouring system components.

From (4), it can already be seen that the first few elements of NMR chain predominantly contribute to $F_{overall}$. This effect is particularly pronounced if the first elements have only loss (i.e., G<1), which deteriorates *F*. On the contrary, inclusion of lossy components close to the chain end can be negligible if the gains of the active preceding elements are high enough (G≫1) and the associated values of *F* are low (e.g., a low-noise pre-amplifier).

### 2.4. Noise Model of NMR Receiving Chain

**Figure 3:**
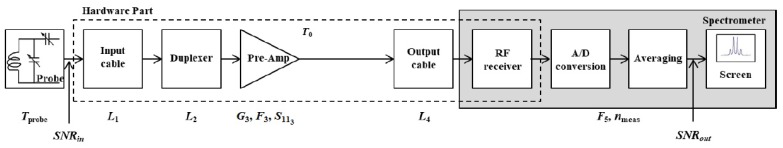
Block diagram of a NMR spectroscopy receiving chain with a single-stage pre-amplifier.

A block diagram of the analysed chain is shown in Figure 3, where $L_{1}$, $L_{2}$, and $L_{4}$ stand for the linear values of power loss for the input cable, duplexer, and output cable, respectively. Furthermore, $G_{3}$ and $F_{3}$ stand for the linear value of power gain and noise figure of the pre-amplifier, respectively. $F_{5}$ stands for the linear value of the spectrometer’s overall noise figure (its RF receiver, A/D converter, and digital signal processor (DSP) altogether). Inserting the above definitions for various chain parameters into (4), one gets the expression for $F_{overall}$ of the NMR receiving chain:

(5) $$\begin{array}{c} F_{{\mathrm{NMR}}_{Rx}}=F_{1}+\frac{F_{2}-1}{G_{1}}+\frac{F_{3}-1}{G_{1}G_{2}}+\frac{F_{4}-1}{G_{1}G_{2}G_{3}}+\frac{F_{5}-1}{G_{1}G_{2}G_{3}G_{4}}, \end{array}$$

which can be rearranged into:

(6) $$\begin{array}{c} F_{{\mathrm{NMR}}_{Rx}}=L_{1}+L_{1}\left(L_{2}-1\right)+L_{1}L_{2}\left(F_{3}-1\right)+\frac{L_{1}L_{2}\left(L_{4}-1\right)}{G_{3}}+\frac{L_{1}L_{2}L_{4}\left(F_{5}-1\right)}{G_{3}}. \end{array}$$

In the last step, we have taken into account that the passive lossy elements of the chain (input cable, duplexer, and output cable) have their input and output impedance matched to $Z_{0}$. Their gains and noise figures are then related by $G_{i}=\frac{1}{L_{i}}$ and $F_{i}=L_{i}$ [5], where *i* stands for the *i*th element of the chain. The losses and gains can be measured using a vector network analyser (VNA), which is often found in NMR laboratories, by measuring the scattering $S_{21}$ parameter of every element [12]. On the other hand, noise figures of any element can be measured by a noise-figure meter [13]; a standard instrument widely used in RF engineering, but not often found in NMR laboratories. A particular problem, in this regard, is the determination of the spectrometer’s noise figure, and a simple approximate method for this is given in the appendix. An alternative determination route is to use the noise figure values found in associated datasheets.

**Figure 4:**
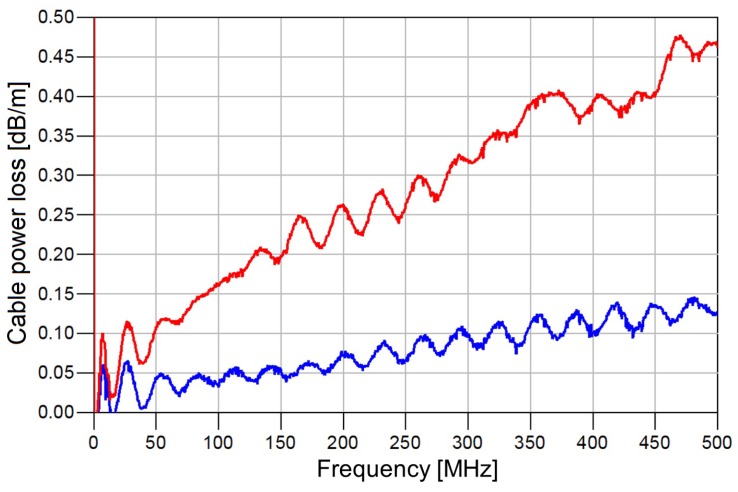
Losses of coaxial cables per unit length in the case of standard coaxial cable (red) and high-quality coaxial cable produced by Fujikura company (blue).

**Figure A1:**
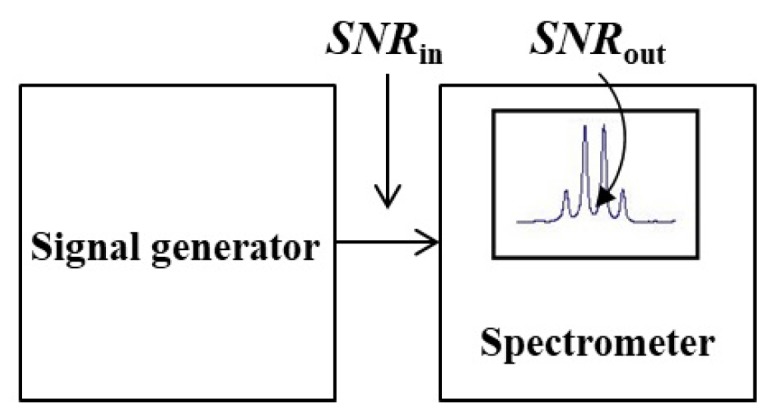
Spectrometer’s overall noise figure measurement: the block diagram.

As the first element in the NMR receiving chain is the cable (Figure A1) that connects the probe output with a duplexer, its noise properties limit the minimum value of $F_{NMR_{Rx}}$ (6). No hardware nor software improvements can decrease it below the value of cable losses. Therefore, to achieve a lower value of $F_{NMR_{Rx}}$ (and, therefore, to increase sensitivity), it is necessary to completely remove the input cable, or at least to minimize it. Figure 4 compares the normalized loss of a standard co-axial cable (in dB/m) that was previously used in our NMR lab to the loss of a high-quality co-axial cable which we obtained during this study. It can be seen that a simple cable replacement immediately improved the noise figure by between 0.05 dB and 0.3 dB, depending on the operating frequency. Here, “ripples” in transfer characteristics are caused by inherent small reflections (impedance mismatch) which always occur in practice.

As we already mentioned, (6) is only a general form where we haven’t taken into account possible impedance mismatches and temperatures which are not equal to $T_{0}$. We will now describe and explain each of these corrections.

### 2.5. Preamplifier’s Impedance Mismatch

If some chain elements have input/output impedances different than $Z_{0}$, signal reflections will occur, causing an effective deterioration of the noise figure of the element itself and the entire RF system. In the NMR receiving chain (Figure 3), there are two elements that may cause notable impedance mismatch: The pre-amplifier and the spectrometer. The spectrometer is basically a special RF receiver, which is constructed very carefully and where great efforts were put in towards good matching. Thus, it is assumed that spectrometer input is perfectly matched to $Z_{0}$ and the possible, but small, mismatch is neglected in our analysis. The pre-amplifier, however, is not always matched to $Z_{0}$. During the construction of a NMR pre-amplifier, three conditions need to be met: (i) The power gain should be the highest possible, (ii) its noise figure should be the lowest possible, and (iii) it has to be matched as close as possible to $Z_{0}$. In addition, the pre-amplifier should be stable (i.e., not prone to unwanted self-oscillations) and have a high dynamic range. Unfortunately, it is impossible to meet all these conditions at the same time, and some trade-off is necessary. To take this into account, we will consider a realistic situation where the elements connected to pre-amplifier’s input and output are well matched to $Z_{0}$, meaning that the signal is not going to reflect back from them, while the pre-amplifier is only mismatched at its input. Thus, its gain and noise figure, corrected due to impedance mismatch [5], are given by [4]:

(7) $$G_{corr.}=G(1-{\left|S_{11}\right|}^{2}),\;\mathrm{and}$$

(8) $$F_{corr.}=1+\frac{F-1}{1-{\left|S_{11}\right|}^{2}},$$

where $S_{11}$ effectively stands for the linear value of input reflection coefficient, which can also be measured by VNA [12]. Taking all this into consideration, the NMR receiving chain noise figure can be expressed as:

(9) $$\begin{array}{c} F_{{\mathrm{NMR}}_{Rx}}=L_{1}+L_{1}\left(L_{2}-1\right)+\frac{L_{1}L_{2}\left(F_{3}-1\right)}{1-{\left|S_{11_{3}}\right|}^{2}}+\frac{L_{1}L_{2}\left(L_{4}-1\right)}{G_{3}\left(1-\right|{\left|S_{11_{3}}\right|}^{2})}+\frac{L_{1}L_{2}L_{4}\left(F_{5}-1\right)}{G_{3}(1-{\left|S_{11_{3}}\right|}^{2})}. \end{array}$$

### 2.6. Non-Standard Temperature of the Probe

In modern NMR measurements of materials in condensed matter physics, the vast majority of measurements are done with both the probe and the sample placed in the environment of very low temperatures (as low as on the order of 100 mK). Hence, (9) should be corrected, as the noise temperature at the input differs significantly from $T_{0}$. Considering the fact that, at standard temperatures and magnetic fields, the noise power changes linearly with temperature [4], it can be shown that the non-standard noise figure of an RF element at a temperature $T\neq T_{0}$ can be expressed by a standard noise figure, as [14]:

(10) $$F=1+\frac{T_{0}}{T}\left(F_{0}-1\right).$$

Before we introduce this correction to (9), it is first necessary to determine the temperature *T*. The temperatures of both the coil and the sample are equal to $T_{coil}$, while the temperature at the NMR probe output equals $T_{0}$. As co-axial cables are usually made from standard metallic materials that are good heat conductors, and as the temperature $T_{coil}$ does not vary during the measurement, there will be a temperature gradient from one cable end to another. This can be approximated by a linear gradient, enabling definition of the average probe temperature:

(11) $$T_{avg}=\frac{T_{coil}+T_{0}}{2}.$$

The approximation of a linear gradient has been tested; it introduces only a negligible uncertainty into our calculations. In addition, this adjustment only shifts the standard noise figures values due to the difference between input noise level at $T_{0}$ and at *T*. This does not affect our conclusions, nor the methodology for improvement of the NMR receiving chain noise properties. So, we can write (9) as:

(12) $$F_{{\mathrm{NMR}}_{Rx}}=1+\frac{2\cdot T_{0}}{T_{coil}+T_{0}}\left[L_{1}+L_{1}\left(L_{2}-1\right)+\frac{L_{1}L_{2}\left(F_{3}-1\right)}{1-{\left|S_{11_{3}}\right|}^{2}}+\frac{L_{1}L_{2}\left(L_{4}-1\right)}{G_{3}(1-{\left|S_{11_{3}}\right|}^{2})}+\frac{L_{1}L_{2}L_{4}\left(F_{5}-1\right)}{G_{3}(1-{\left|S_{11_{3}}\right|}^{2})}-1\right].$$

### 2.7. Signal Averaging

As it is well-known, the averaging of multiple measurements can improve effective SNR drastically (under the assumption that the physical properties of the NMR system do not vary during averaging). Due to the stochastic nature of noise [15], the output voltage SNR increases by the square root of the number of averaged measurements ($n_{meas}$); that is, the power SNR increases with $n_{meas}$ [16]: ${\mathrm{SNR}}_{avg}=n_{meas}\cdot {\mathrm{SNR}}_{single}$, where ${\mathrm{SNR}}_{single}$ and ${\mathrm{SNR}}_{avg}$ stand for the SNR of the single and averaged measurements, respectively. As the noise figure (3) is defined as the ratio of input and output SNRs, it is clear that the output SNR is enhanced by $n_{meas}$, while, at the same time, the effective noise figure decreases linearly:

(13) $$\begin{array}{c} F_{avg}=\frac{{\mathrm{SNR}}_{in}}{{\mathrm{SNR}}_{out_{avg}}}=\frac{{\mathrm{SNR}}_{in}}{{\mathrm{SNR}}_{out_{single}}\cdot n_{meas}}=\frac{F_{single}}{n_{meas}}, \end{array}$$

where $F_{single}$ and $F_{avg}$ stand for noise figures of single and averaged measurements, respectively. Finally, the NMR receiving chain noise figure is expressed as:

(14) $$\begin{array}{cc} F_{{\mathrm{NMR}}_{Rx}}=\frac{1}{n_{meas}} & \left\{1+\frac{2\cdot T_{0}}{T_{coil}+T_{0}}[L_{1}+L_{1}\left(L_{2}-1\right)+\frac{L_{1}L_{2}\left(F_{3}-1\right)}{1-{\left|S_{11_{3}}\right|}^{2}}+\right.\\ & \left.\frac{L_{1}L_{2}\left(L_{4}-1\right)}{G_{3}(1-{\left|S_{11_{3}}\right|}^{2})}+\frac{L_{1}L_{2}L_{4}\left(F_{5}-1\right)}{G_{3}(1-{\left|S_{11_{3}}\right|}^{2})}-1]\right\}. \end{array}$$

### 2.8. The Case of a Two-Stage Preamplifier

**Figure 5:**
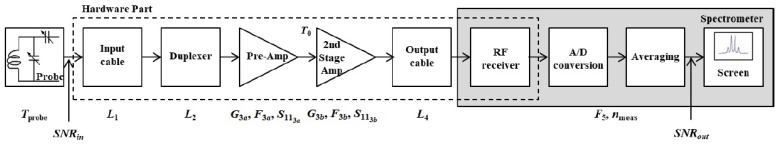
Block diagram of NMR spectroscopy receiving chain for a two-stage preamplifier.

We will also consider the case of a two-stage pre-amplifier (Figure 5), as we will use it later. Equation (14) can be expanded to (15), where the indices are the same as in (14), except, now, the index ‘3b’ refers to the second stage of the two-stage pre-amplifier:

(15) $$\begin{array}{c} F_{{\mathrm{NMR}}_{Rx}}=\frac{1}{n_{meas}}\left\{1+\frac{2\cdot T_{0}}{T_{coil}+T_{0}}[L_{1}+L_{1}\left(L_{2}-1\right)+\frac{L_{1}L_{2}\left(F_{3a}-1\right)}{1-{\left|S_{11_{3a}}\right|}^{2}}+\frac{L_{1}L_{2}\left(F_{3b}-1\right)}{G_{3a}(1-{\left|S_{11_{3a}}\right|}^{2})(1-{\left|S_{11_{3b}}\right|}^{2})}+\right.\\ \left.\frac{L_{1}L_{2}\left(L_{4}-1\right)}{G_{3a}(1-{\left|S_{11_{3a}}\right|}^{2})G_{3b}(1-{\left|S_{11_{3b}}\right|}^{2})}+\frac{L_{1}L_{2}L_{4}\left(F_{5}-1\right)}{G_{3a}(1-{\left|S_{11_{3a}}\right|}^{2})G_{3b}(1-{\left|S_{11_{3b}}\right|}^{2})}-1]\right\}. \end{array}$$

## 3. Experimental Verification of Developed Noise Model

### 3.1. General Approach

To test the derived expression for the NMR receiving chain noise figure ((14) and (15)) experimentally, we performed multiple measurements on two different samples (and, more importantly, different nuclei) with a measurement setup where different pre-amplifiers (with different gain and noise properties) were used. Clearly, the pre-amplifier is the only active element in the chain (besides the spectrometer) and, as such, variation of its parameters will result in the largest change in recorded signal quality (i.e., SNR). As different frequencies were tested, appropriate duplexers were also used, but both were based on the same technology and had equivalent losses. The remainder of the setup (cables, spectrometer, and NMR probe) were left the same. We used a Tecmag Apollo spectrometer and a commercial NMR probe.

By measuring the characteristics of the individual elements of the chain, and by using (14) and (15), we calculated the noise figures of the receiving chain in every measurement of the test. Then, we performed a set of measurements on one sample and calculated $SNR_{in}$. If our noise figure calculations were correct, we should always obtain the same value of $SNR_{in}$, since we only changed one or two elements in the receiving chain and not the source. This will show the inner consistency of our analysis. Our $SNR_{in}$ value was, then, compared to ${\mathrm{SNR}}_{in}^{0}$ (determined from (1) and (2)), which should be similar and, thus, prove the validity of our approach.

### 3.2. Measurements Description

Our first measurement was on the ^63^Cu signal in SeCuO_3_ (two sets of measurements) [17], while the second one was the ^133^Cs signal in Cs_2_Cu_2_SnF_12_ (three sets of measurements) [18]. A full description of the measurement setup is available in Table 1, while the NMR properties of the measured nuclei are presented in Table 2. We will focus on effects of different pre-amplifiers in the measurement setup, as there are a vast number of commercially available types and as it is the only active element (besides the spectrometer). While choosing the pre-amplifier, one should bear in mind that its characteristics will have a significant impact on the overall noise figure (because G≫1) and decrease the contribution of losses accumulated up to its output.

Before we present the data, we would like to describe several properties of NMR signals that will help to understand their behaviour and role in signal intensity. We have already mentioned these at the beginning of the paper: Quadrupolar splitting, line-width, spin–spin relaxation time, and isotope abundance. The latter is, perhaps, the easiest to explain. In nature, every element can be found in several isotopes. These have different nuclear properties, such as the gyromagnetic constant γ which defines our measurement frequency. They can also be found in different relative abundances, such as ^63^Cu and ^65^Cu, whose abundance ratio equals 0.691:0.309. So, by recording an NMR signal of ^63^Cu, the signal intensity will be only approximately 69% of all the copper nuclei in the sample. Hence, we need to keep the ratio of isotopes in mind when calculating what signal size we expect to see.

Line-width is, as the name indicates, the width of the NMR line. It is defined by the local homogeneity of the crystal in the vicinity of the measured nuclei, and by the homogeneity of the external magnetic field. If these were perfectly homogeneous, our NMR signal could be described by a Dirac delta function $\delta (\omega -\omega_{0})$ of infinitesimal width. In reality, neither the samples nor the magnetic field are ideally homogeneous. This will become manifest, such that our NMR frequency will be defined by a Lorentzian (or Gaussian) function of finite width. As the spectral width is preserved, signal amplitude will proportionally drop to compensate for it.

**Figure 6:**
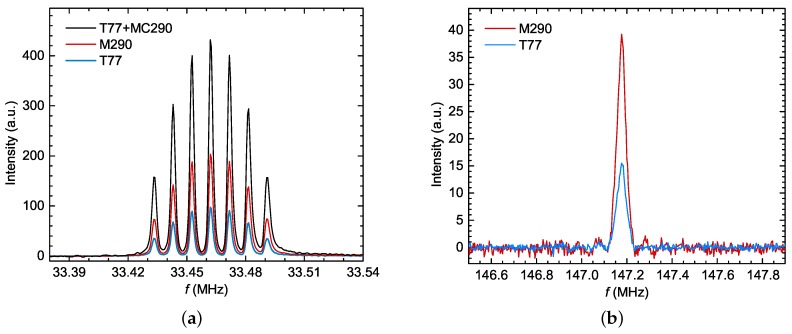
NMR spectra measured to check the validity of NMR receiving chain’s noise figure calculation: (**a**) ^133^Cs in Cs_2_Cu_3_SnF_12_ (the splitting of the spectra due to quadrupolar splitting into 7 lines is visible), (**b**) central line of ^63^Cu in SeCuO_3_ (quadrupolar satellites are too far apart to be excited by a single excitation pulse).

Quadrupolar splitting appears with nuclei of spin I>1/2, in which case, the nucleus is sensitive to the direction of the local electrostatic gradients. Unless it is located at a very symmetrical position, our NMR signal will split into 2·I lines of well-defined intensities [3,7], since it will sense the local distribution of the electric potential. A textbook example is shown in Figure 6a, where the ^133^Cs signal has been split into seven lines. Again, as the spectral weight is preserved, the split signal amplitude will drop from the “unsplit case”. The size of quadrupolar splitting can vary from a few kHz (as for the ^133^Cs signal) to several tens of MHz (as for the ^63^Cu signal).

The spin–spin relaxation time ($T_{2}$) is a measure of how rapidly in time the NMR signal drops, from the moment the nucleus is excited to the moment the NMR signal is recorded. Normally, it follows an exponential $e^{-\frac{t}{T_{2}}}$ dependence, and is determined by the properties of the sample. The time of measurement of the signal, *t*, is set by the dead-time of electrical discharge of the coil after the pulse.

The two pre-amplifiers used for the ^63^Cu measurements were the MITEQ AU-1114-SMA [19] (abbreviated as M290) and the THAMWAY N141-206AA(D) [20] (abbreviated as T77). The first pre-amplifier operated at $T_{0}$, while the second one was cooled to the temperature of liquid nitrogen (77 K). Both were used as single-stage pre-amplifiers. On the other hand, for the ^133^Cs measurements, these two units were used as single-stage pre-amplifiers, but the T77 was also used as the first stage of a two-stage pre-amplifier, with a Mini-Circuits HELA -10D+ [21] (abbreviated as MC290) used as the second stage. The MC290 operated at $T_{0}$.

### 3.3. Results

The experimental verification results are shown in Table 3, and the measured data are shown in Figure 6.

From the two measurements of ^63^Cu in SeCuO_3_ (i.e., the associated $SNR_{out}$), it follows that the values of NMR receiving chain $SNR_{in}$ were 12.17 dB and 12.14 dB for M290 and T77, respectively. The difference between the predicted values was around 0.03 dB, which is comparable to measurement uncertainty. The evaluated value of the same SNR from (1) and (2) gave 15.20 dB. Here, we took into account that the signal amplitude was reduced, due to the short $T_{2}$ time (to 75% of the value), broadened line-width, NQR splitting of spectral lines (to 33% of the value), number of crystallographic sites (to 50% of the value), and abundance of the ^63^Cu isotope (69%). Out of these, the most ambiguous parameter was line broadening, because it could not be estimated with high precision. However, even if we conservatively estimate that the spectral weight was reduced to 10 % of its value, our $SNR_{in}$ estimation, using (1) and (2), was of an acceptable order of magnitude. To keep this estimate simple, we did not discuss the dependence of intensity on the orientation of the sample (i.e., orientation of the quadrupolar principle value with respect to external field), or NMR coil, but these effects would further reduce the signal intensity and, thus, make our result match even better.

The calculated values of the NMR receiving chain $SNR_{in}$ for measurements of ^133^Cs in Cs_2_Cu_3_SnF_12_, as shown in Figure 6a, where the M290 and T77 pre-amplifiers were used, were equal to 37.54 dB and 37.55 dB, respectively; again, showing a good consistency of the results. The evaluated value of the NMR receiving chain $SNR_{in}$, from (1) and (2), in this case, was 35.31 dB, which was adjusted only for the NQR splitting of spectral lines (to 25% of the value), as the line-width was only approximately 5kHz (FWHM). In this system, there was only one crystallographic site, the abundance of the ^133^Cs isotope was 100%, and the $T_{2}$ time did not show any considerable effect. Therefore, the validity of the derived expression is confirmed in this case, as well.

We can now discuss the temperature effect of the co-axial cable in the NMR probe (11). If we, conservatively, take that the cable temperature was $T_{coil}$, then all of the calculated results in Table 3 should be decreased by 0.6–1.0 dB. In another conservative limit, if the cable temperature was taken as $T_{0}$, then all calculated results should be increased by the same amount. Therefore, the uncertainty of the co-axial cable’s average temperature was well within the accuracy of our analysis and its comparison to the estimated result using (1) and (2).

From the measured values presented in Table 3, it can be seen that, in both cases, the value of $SNR_{out}$ was higher in the case of M290, regardless of the fact that T77 had a lower noise factor at 77 K. This is because the amplifier did not reduce overall noise factor as much as it did the gain (Figure 2). An improvement would have been achieved if the gain value of T77 had also been approximately 36 dB. To analyse this situation in more detail, we wanted to see what the effect of increasing the gain of the T77 unit artificially would be, by adding a second amplifier in a cascade after the T77, thus, creating a two-stage pre-amplifier with an overall (measured) gain of 37.88 dB and noise figure of 1.25 dB.

This was done using a MC290, as mentioned earlier. From Figure 6a, it can be seen that this resulted in a signal amplitude that was, indeed, larger than that of the M290, but did not result in a better $SNR_{out}$. In this case, the predicted value of the NMR receiving chain $SNR_{in}$ was 36.24 dB. The difference between this value and those from the two previous measurements was around 1.3 dB, which was a bit larger than our previous results (16% on the voltage scale). This error appears because (15) is somewhat more imprecise than (14) for a single-stage pre-amplifier. Namely, in the derivation of (14) for a single-stage pre-amplifier chain, we assumed that there was no signal reflection at pre-amplifier output, due to the next chain element being matched to $Z_{0}$. However, in the case of a two-stage pre-amplifier, the second stage amplifier input was not matched to $Z_{0}$, causing multiple reflections of the signal between the two stages. To take this into account, both the amplitude and phase of all four *S* parameters of both amplifiers should be measured and input into (14). However, this would make the analysis much more complex and only slightly more precise. Therefore, for the sake of clarity, simplicity, and to offer the NMR community an easy way to calculate the properties of a NMR receiving chain, we will keep (15) as it is, as a good pragmatic result.

Now, we can draw some important conclusions. First of all, when building an NMR setup, it is necessary to carefully choose appropriate elements, with respect to the existing ones. For example, when choosing the pre-amplifier, focusing on the one with the lowest noise figure alone might not be the best solution. It can be seen, in Table 1 and Table 3, that, although the T77 had a significantly lower noise figure than the M290, the $SNR_{out}$ value of the NMR receiving chain was notably higher with the use of M290, because its power gain is much higher than the gain of the T77 and the impedance matching of the M290 is much better than that of the T77. This is why it is necessary to consider all three pre-amplifier values—power gain, noise figure, and impedance matching (or its input reflection coefficient)—and select those with optimum values. Additionally, since the co-axial cable connecting the NMR coil and the duplexer and the duplexer itself are in front of the pre-amplifier, these two elements need to be of the highest quality.

The results of our two-stage amplification raises the question what type of amplifier would give better $SNR_{out}$ than the commercial unit M290. For this purpose, we will use (14) to predict the receiving chain $SNR_{out}$ values for both measured samples, in the case of using a hypothetical state-of-the-art NMR pre-amplifier (abbreviated as SOA), which has a power gain and noise figure of 40 dB and 0.3 dB, respectively, and is perfectly matched to $Z_{0}$. The prediction results are shown in Table 4.

As M290 resulted in the greatest $SNR_{out}$ for measurements of both samples, the SOA was compared with this unit. So, for the same $SNR_{in}$ of ^63^Cu in SeCuO_3_, the receiving chain with SOA generated a $SNR_{out}$ 2.16 dB greater than that of M290. This corresponds to a 65% enhancement in linear scale with SOA (i.e., to reach equal $SNR_{out}$, SOA would, in our configuration, measure 1.65 times faster). This is a very significant decrease in measurement time. Furthermore, for ^133^Cs measurements in Cs_2_Cu_3_SnF_12_, this difference rose to 2.76 dB in logarithmic, or 89% in linear scale. Here, the measurement time was reduced by almost two times, which is a drastic decrease. These two predictions prove that pre-amplifier properties, along with those of the spectrometer, are one of the most important aspects of a NMR system.

**Figure 7:**
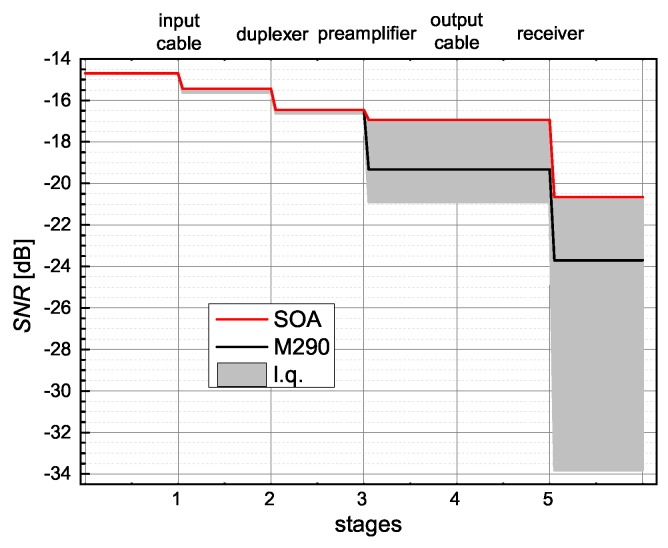
Calculated SNR deterioration of the weakest signal that has ${\mathrm{SNR}}_{\mathrm{in}}=-14.7$ dB along NMR receiving chain, for various conditions: existing chain with SOA (red) or M290 (black). The gray area depicts an estimated range of NMR signal’s SNR deterioration by placing low-quality (l.q.) elements in the chain.

We can, also, consider a case of an extremely low NMR signal and extract a theoretical limit for measurement time shortening. Let us consider the case of a metallic sample in single crystal form. The idea is to study its crystal properties and, therefore, the sample cannot be powdered. However, since it is metallic, the sample must be made thin, so that the coil filling factor remains as close to 1 as possible, and is not reduced by a finite penetration depth. Let us analyse the signal that was measured at 10 K, and which had a $SNR_{out}$ value of 24.08 dB after 60,000 acquisitions. If we used the M290 and kept the properties of the rest of the setup the same, the $SNR_{in}$ would be equal to −14.71 dB. If we used SOA, it would lead to a $SNR_{out}$ value of 27.12 dB under the same measurement conditions (i.e., a 3.03 dB difference). Hence, the measurement time could be shortened to half with additional system enhancements. In Figure 7, we show, in detail, how the SNR changes through the chain for such a weak signal, in the case of M290 and SOA. In addition, the graph shows how the SNR would deteriorate in the case of low quality (l.q.) chain components. This can, of course, cover a broad spectrum of poor performance, but the worst-case scenario (bottom edge of the grey area) was calculated using: $L_{1}=0.5$ dB, $L_{2}=0.6$ dB, $G_{3}=26$ dB, $F_{3}=3$ dB, $S_{11_{3}}=-13$ dB, $L_{4}=0.7$ dB, and $F_{5}=40$ dB.

## 4. Improvement Suggestions

We can, now, discuss how the NMR community can benefit from the presented analysis, and also point out ways in which all elements of NMR setups can be further developed.

From earlier discussions and (14), it is apparent that the cable at the beginning of the receiving chain (between the NMR probe and duplexer) should be as short as possible. If the cryostat and spectrometer are necessarily distant from each other, and long cables are required to connect them, then it is imperative to use the shortest and the least noisy cable (i.e., the cable with the lowest losses) between the NMR probe and duplexer [22]. In this way, the noise properties of elements after the pre-amplifier are efficiently suppressed, while those of the pre-amplifier and elements before are negligible.

Accordingly, enhancements made on noise properties of the duplexer and pre-amplifier will affect the overall noise properties the most. Duplexer noise properties can directly be modified by, for example, cryogenic cooling [23], just as can be done with the pre-amplifier. Otherwise, the duplexer power loss can be decreased by using microwave substrates with high relative permittivity [24], extremely low losses and a conductive metallic layer, the use of ultra-fast and ultra-low-loss switching diodes, the use of low-loss connectors, and so on. When dealing with pre-amplifier properties, on the other hand, one needs to take into consideration the conditions described in Section 2.5. It is impossible to find a pre-amplifier that has all conditions met perfectly; thus, a pre-amplifier with optimum properties at corresponding frequencies of NMR measurements needs to be found. However, even state-of-the-art duplexers and pre-amplifiers cannot enhance the receiving chain’s overall noise properties if the noise properties of the elements up to that point are poor. For example, the NMR coil and its tuning/matching network should always be connected directly to a co-axial cable which connects them to NMR probe output. A connection by non-insulated wire must be avoided, as this will lead to much worse noise properties in the NMR response signal, even before the signal reaches the environment of $T_{0}$. Here, poor noise properties will dominate the rest of the network and improving the noise properties of the rest of the chain will not solve the problem.

As Table 1 clearly shows, the spectrometer’s noise properties are, by far, the worst in the receiving chain. However, the spectrometer’s noise figure impact on system performance can be decreased by a single-stage pre-amplifier. Such a pre-amplifier should have a high enough power gain, such that the $F_{5}/G_{3}$ ratio in (14) is suppressed as much as possible. If a high-gain single-stage pre-amplifier is not available, an additional amplifier can be added (in a cascade with the initial one) to compensate for the insufficient power gain level. The two pre-amplifiers should be arranged such that the unit with the lower noise figure is farther from spectrometer, in order to accomplish better noise properties [5]. However, although a pre-amplifier cascade solves the problem, it needs to be done carefully, such that the cascade’s output signal does not exceed the spectrometer’s maximum input level.

The only non-hardware part of the NMR receiving chain which is directly taken into account in (14) is the averaging of multiple measurements. Although this method is quite efficient, it can still be improved. If it is combined with different sorts of digital filtering, additional enhancement of the chain’s overall noise properties can be achieved [25]. However, averaging has its boundaries. The number of measurements can be increased only up to a point where the method reaches saturation [26]. This is why it is necessary to combine hardware-based methods with digital filtering, in order to shift the saturation point up to a higher number of measurements.

The last comment regarding the derived NMR receiving chain noise figure expression is an explanation of its possible values. While it is considered that a cascade noise figure value scan only be higher than 1 (i.e., 0 dB), this is only true if only hardware elements are being considered. As (14) contains averaging, which is the chain’s non-hardware part, it is possible to achieve values of the noise figure lower than 1 (i.e., lower than 0 dB).

Finally, the authors want to point out that a Python program which calculates the noise figure and predicts the input and output SNRs of the NMR receiving chain, using (14), (15) and (3), from user-input values of the receiving chain elements of their NMR spectroscopy system, is available on GitHub [27].

## 5. Conclusions

We have developed a simple noise model for a NMR receiving chain and verified it experimentally. The difference between the predicted and obtained values was lower than 0.1%. With the help of our developed model, we presented different ways to decrease the overall NMR measurement time, with a possible decrease value of almost 90%. The elements of the NMR receiving chain that affect the system’s overall noise properties (and, therefore, its measurement sensitivity) the most have been discussed, thus paving the way for future research into the development of NMR systems. A Python program that calculates noise properties of a chain, using the noise properties of individual elements, has been created and made available online for the NMR community.

## Figures and Tables

**Table 1:** Electric parameters of receiving chain elements used in both sets of measurements.

Compound		SeCuO_3_	Cs_2_Cu_3_SnF_12_
Parameter	Symbol	Values	
Measurement frequency (MHz)	-	147.20	33.50
Coil and sample temperature (K)	$T_{coil}$	20	30
DC magnetic field (T)	$B_{0}$	11.90	6
Input cable loss (dB)	$L_{1}$	0.28	0.10
Duplexer loss (dB)	$L_{2}$	0.27	0.43
M290 gain (dB)	$G_{3}$	36.13	36.52
M290 noise figure (dB)	$F_{3}$	1.11	1.14
M290 reflection coefficient (dB)	$S_{11_{3}}$	−16.43	−13.50
T77 gain (dB)	$G_{3}$, $G_{3a}$	28.54	27.89
T77 noise figure (dB)	$F_{3}$, $F_{3a}$	0.32	1.07
T77 reflection coefficient (dB)	$S_{11_{3}}$, $S_{11_{3a}}$	−7.57	−8.05
MC290 gain (dB)	$G_{3b}$	-	10.75
MC290 noise figure (dB)	$F_{3b}$	-	4.22
MC290 reflection coefficient (dB)	$S_{11_{3b}}$	-	−27.58
Output cable loss (dB)	$L_{4}$	0.46	0.62
Spectrometer noise figure (dB)	$F_{5}$	33.50	38.40
Number of measurements	$n_{meas}$	200	400
Input impedance M290 (Ω)	$Z_{in}$	51.20	72.80
Output impedance M290 (Ω)	$Z_{out}$	54.50	45.60
Input impedance T77 (Ω)	$Z_{in}$	45.77	34.70
Output impedance T77 (Ω)	$Z_{out}$	101.46	38.20
Input impedance MC290 (Ω)	$Z_{in}$	50	50
Output impedance MC290 (Ω)	$Z_{out}$	50	50

**Table 2:** NMR properties of the nuclei used in experiments.

Compound	Nucleus	γ (MHz/T)	Spin	Abundance (%)	Quadrupole Splitting
SeCuO_3_	^63^Cu	11.285	3/2	69.1	48.05 MHz
Cs_2_Cu_3_SnF_12_	^133^Cs	5.5844	7/2	100	9.54 kHz

**Table 3:** Results of experimental verification of the derived expression for NMR spectroscopy receiving chain.

Compound	SeCuO_3_	Cs_2_Cu_3_SnF_12_
Frequency (MHz)	147.20	33.50
**M290**		
Measured $SNR_{out}$ (dB)	30.11	56.00
Determined $F_{NMR_{Rx}}$ (dB)	−17.95	−18.46
Calculated $SNR_{in}$ via (14) and (3) (dB)	12.17	37.54
**T77**		
Measured $SNR_{out}$ (dB)	24.97	48.32
Determined $F_{NMR_{Rx}}$ (dB)	−12.83	−10.77
Calculated $SNR_{in}$ via (14) and (3) (dB)	12.14	37.55
**T77 with MC290**		
Measured $SNR_{out}$	-	55.65
Determined $F_{NMR_{Rx}}$ (dB)	-	−19.41
Calculated $SNR_{in}$ via (15) and (3) (dB)	-	36.24
**Calculated** ${\mathrm{SNR}}_{\mathrm{in}}$ **via (1) (dB)**	15.20	35.31

**Table 4:** Results of $SNR_{in}$ prediction of NMR receiving chain using SOA.

Compound	SeCuO_3_	Cs_2_Cu_3_SnF_12_
Frequency (MHz)	147.20	33.50
$SNR_{in}$ (dB)	12.17	37.54
$SNR_{out}$ using M290 (dB)	30.11	56.00
Predicted $SNR_{out}$ using SOA (dB)	32.28	58.77
$SNR_{out}$ enhancement (dB)	2.16	2.76
$SNR_{out}$ enhancement (%)	65	89

**Figure A2:**
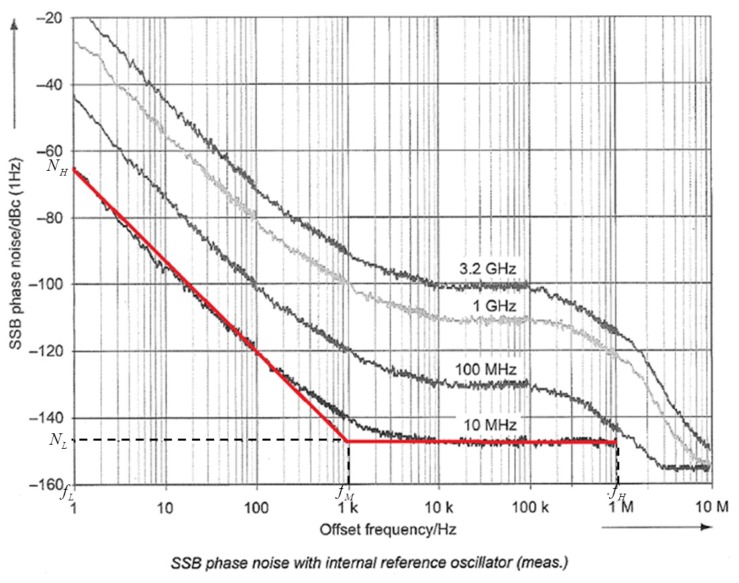
RF generator’s $N_{SSB}-f$ chart and linear approximation of its curve (red line). The figure corresponds to Rohde & Schwarz SMC100A generator (with permission from Rohde & Schwarz).

## Appendix A. Spectrometer Noise Figure Determination

At its core, a spectrometer is an RF receiver that does complex processing of a measured NMR signal before the results can be properly shown on its screen. Its typical processing blocks include analog filtering, pre-amplification, down-converting to an intermediate frequency, gain control, A/D conversion, digital quadrature detection, digital filtering, fast Fourier transformation, and averaging. Each of these processes is done within an electronic device that injects its own noise into the system, causing deterioration of the overall noise properties. So, to calculate spectrometer noise properties, one would need to know a detailed noise model of each of its elements. In practice, this information is never available to the user. Fortunately, it is possible to describe the spectrometer as a “black box” with its own effective noise figure. This enables a simple measurement of spectrometer’s noise figure.

In order to determine the spectrometer’s overall noise figure, one needs to connect a RF generator to spectrometer’s input (Figure A1), analyse the signal at the spectrometer’s output (its screen), calculate SNRs at both the spectrometer’s input and output, and then use (3) to calculate the noise figure. It is presumed that a high-quality synthesized RF generator is at the user’s disposal.

To calculate $SNR_{out}$, both the output voltage signal amplitude, $A_{S_{out}}$, and noise RMS voltage value, $V_{RMS_{out}}$, need to be determined:

(A1) $${\mathrm{SNR}}_{out}\left[\mathrm{dB}\right]=20\cdot \log\left(\frac{A_{S_{out}}\left[V\right]}{V_{RMS_{out}}\left[V\right]}\right).$$

Furthermore, to calculate the value of $SNR_{in}$, one needs to analyse the single sideband (SSB) phase noise of the RF generator connected to the spectrometer’s input (as phase noise is the dominant noise type in synthesized RF signal generators). This is done with the help of an $N_{SSB}-f$ chart, given in the generator’s datasheet (Figure A2):

To calculate the equivalent net power of a generator’s overall phase noise, one needs to integrate the corresponding curve from $f_{L}$ to $f_{H}$—where $f_{H}$ is equal to one half of frequency bandwidth of spectrometer’s filter (set in the spectrometer’s NMR measurements software), while $f_{L}$ is equal to zero—and then double the calculated result to get the overall phase noise of both side-bands. The easiest way to do this is to approximate the curve with a linear and constant part in logarithmic scale, transfer the approximation to a linear scale, and then integrate the approximation curve from $f_{L}$ to $f_{H}$:

(A2) $$\begin{array}{c} N_{in}\left[\mathrm{dBc}\right]=10\cdot \log\left\{\frac{10^{\frac{N_{H}\left[\mathrm{dBc}\right]}{10}}}{1-\frac{\Delta N[\mathrm{dB}]}{10\cdot \log(f_{M})}}\cdot \left[f_{M}^{\left(1-\frac{\Delta N[\mathrm{dB}]}{10\cdot \log(f_{M})}\right)}-1\right]+10^{\frac{N_{L}\left[\mathrm{dBc}\right]}{10}}\cdot \left(f_{H}-f_{M}\right)\right\}+3, \end{array}$$

where $N_{H}$ and $N_{L}$ stand for highest and lowest value of phase noise, $\Delta N\left[\mathrm{dB}\right]=N_{H}\left[\mathrm{dBc}\right]-N_{L}\left[\mathrm{dBc}\right]$, and $f_{M}$ stands for the breaking frequency between the linear and constant parts of the approximation curve. The unit of input noise calculated in (A2) is dBc, which stands for decibels relative to the carrier. This unit describes noise power level relative to power level of generated RF signal. So, for example, if noise equals −50 dBc and signal equals 0 dBm, the noise is then equal to −50 dBm; if the signal equals −50 dBm, the noise is then equal to −100 dBm (where dBm stands for decibels relative to 1 mW, which is clearly a unit of power). Taking this into consideration, it can be seen that $SNR_{in}$ is constant, with its logarithmic value equal to the negative value of (A2):

(A3) $${\mathrm{SNR}}_{in}\left[\mathrm{dB}\right]=-N_{in}\left[\mathrm{dBc}\right]=-10\cdot \log\left\{\frac{10^{\frac{N_{H}\left[\mathrm{dBc}\right]}{10}}}{1-\frac{\Delta N[\mathrm{dB}]}{10\cdot \log(f_{M})}}\left[f_{M}^{\left(1-\frac{\Delta N[\mathrm{dB}]}{10\cdot \log(f_{M})}\right)}-1\right]+10^{\frac{N_{L}\left[\mathrm{dBc}\right]}{10}}\left(f_{H}-f_{M}\right)\right\}-3\mathrm{dB}.$$

Finally, combining (3), (A1) and (A3), the spectrometer’s overall noise figure is, then, equal to:

(A4) $$\begin{array}{c} F_{spectrometer}\left[\mathrm{dB}\right]={\mathrm{SNR}}_{in}\left[\mathrm{dB}\right]-{\mathrm{SNR}}_{out}\left[\mathrm{dB}\right]=\\ -10\cdot \log\left\{\frac{10^{\frac{N_{H}\left[\mathrm{dBc}\right]}{10}}}{1-\frac{\Delta N[\mathrm{dB}]}{10\cdot \log(f_{M})}}\;\left[f_{M}^{\left(1-\frac{\Delta N[\mathrm{dB}]}{10\cdot \log(f_{M})}\right)}-1\right]+10^{\frac{N_{L}\left[\mathrm{dBc}\right]}{10}}\cdot \left(f_{H}-f_{M}\right)\right\}-3\mathrm{dB}-20\cdot \log\left(\frac{A_{S_{out}}\left[V\right]}{V_{RMS_{out}}\left[V\right]}\right). \end{array}$$

Of course, one should be aware that (A4) presumes the dominance of the RF generator’s phase noise during measurements. Furthermore, the spectrometer’s overall noise figure depends on the spectrometer’s parameters, such as power gain, frequency bandwidth of its filter, and number of points of current measurement, and on the value of measurement frequency, which is the frequency of the generated sine wave at the spectrometer input. This means that (A4) is a good estimation only for the current set of parameters, but will change if some of these parameters are changed. So, to get the correct value, one needs to measure the spectrometer’s overall noise figure for the exact set of parameters one intends to use in the current NMR measurement.
